# The effects of mind-body exercise on anxiety and depression in older adults: a systematic review and network meta-analysis

**DOI:** 10.3389/fpsyt.2024.1305295

**Published:** 2024-02-07

**Authors:** Yangjian Dong, Xinxin Zhang, Rongting Zhao, Lan Cao, Xiaoqin Kuang, Jiwei Yao

**Affiliations:** ^1^ College of Physical Education and Health, Guangxi Normal University, Guilin, China; ^2^ College of Physical Education, China Three Gorges University, Yichang, China; ^3^ College of Physical Education and Health, Guilin University, Guilin, China

**Keywords:** mind-body exercise, anxiety, depression, older adults, network meta-analysis

## Abstract

**Background:**

Limited research directly compares the clinical effects of different types of mind-body exercises on anxiety and depression in older adults. Therefore, we conducted a systematic review and network meta-analysis of randomized controlled trials that meet the inclusion criteria to explore the intervention effects of five different types of mind-body exercises in improving anxiety and depression in older adults.

**Methods:**

We followed the PRISMA-NMA guidelines and conducted searches in the Web of Science, PubMed, the Cochrane Library, and Embase databases up to July 28, 2023. The language was limited to English. Two independent reviewers conducted literature screening and data extraction. Review Manager 5.4 was used to perform Pairwise meta-analysis and risk assessment, while STATA version 15 software was used for network meta-analysis.

**Result:**

A total of 42 studies, involving 2974 participants, were included. The results of the traditional meta-analysis showed that mind-body exercises were superior to the control group in alleviating anxiety (SMD: -0.87, 95% CI: -1.43, -0.31, p<0.05, I^2^ = 95%) and depressive (SMD: -0.52, 95% CI: -0.71, -0.34, p<0.05, I^2^ = 80%). In the network meta-analysis, the ranking of treatment effects for anxiety showed that Tai Chi > Qigong > Yoga > Dance > control group, while for depression, the ranking showed Tai Chi > Pilates > Yoga > Qigong > Dance > control group.

**Conclusion:**

This study found that mind-body exercises have positive effects on improving anxiety and depression in older adults. Among the five different types of mind-body exercise interventions, Tai Chi was considered an effective approach for improving anxiety and depression. However, we encourage older adults to choose exercise modalities that suit their interests to enhance adherence.

**Systematic review registration:**

http://www.crd.york.ac.uk/PROSPERO/, identifier CRD42023464296.

## 1 Introduction

Anxiety and depression have become one of the most common mental health problems globally, affecting approximately 340 million people (1). Research by Hay et al. indicates that between 2005 and 2015, the prevalence of anxiety increased by 14.9% and the prevalence of depression increased by 18.4% (2). The old adults are the main population affected by anxiety and depression, as they often face challenges such as social isolation and lack of family support, in addition to the threat of declining physical function and chronic health issues (3). Therefore, finding the best treatment measures for anxiety and depression in old adults has become an important scientific issue of global concern. Currently, the main treatment methods for anxiety and depression include medication therapy and cognitive behavioral therapy. However, both of these treatment measures have certain limitations. On one hand, the elderly may have weaker physical health and poor medication adherence, which restricts the use of medication therapy. On the other hand, cognitive behavioral therapy requires face-to-face communication between patients and therapists, which may cause fear, anxiety, and privacy concerns for patients, thereby negatively affecting the mental and physical health of the therapist (4). Additionally, the high cost of treatment also increases the economic burden on patients. Therefore, the current treatment methods for anxiety and depression still need further improvement.

An increasing number of research and commentary reports indicate that mind-body exercise plays a positive role in alleviating anxiety and depression symptoms in older adults. “Mind-body exercise” is a fitness activity that integrates physical and psychological elements. It emphasizes the coordination of body movements and mental processes, aiming to promote physical health while also contributing to mental well-being (5, 6). This exercise typically includes various forms such as yoga, tai chi, Pilates, qigong, and dance. As a complementary and alternative therapy, mind-body exercise is prevalent among older adults due to its ease of learning and low requirements for equipment and space (7–9). For example, yoga has been proven to reduce anxiety and depression levels in older adults, while tai chi exercise also improves their psychological health (10, 11). In addition, relevant meta-analysis results have shown significant therapeutic effects of tai chi in improving anxiety and depression in older adults (12–14). However, there is a wide range of mind-body exercise forms, including differences in exercise frequency, duration, and target population, making it unclear which therapy is more suitable for older adults. Moreover, most randomized controlled trials (RCTs) and traditional meta-analysis methods usually only compare treatment effects between two interventions, without providing a comprehensive comparison of different types of mind-body exercise interventions. The use of traditional pairwise comparison methods has some major limitations.

Therefore, this study chose to use the network meta-analysis (NMA) method to comprehensively compare multiple different intervention measures. Through NMA, we can use the indirect comparison method to quantitatively evaluate the therapeutic effects of different intervention measures in similar situations, thus providing a basis for selecting the best treatment plan. At the same time, we will rank various intervention measures. This study aims to evaluate the impact of different types of mind-body exercise on anxiety and depression symptoms in old adults through network meta-analysis, to provide the best treatment plan for clinical doctors in choosing non-pharmacological treatment options.

## 2 Materials and methods

As this is a systematic review, ethical approval is not required. We have registered this study and network meta-analysis on the International Prospective Register of Systematic Reviews (PROSPERO) with registration number CRD42023464296, and have strictly adhered to the Preferred Reporting Items for Systematic Reviews and Meta-Analyses Network Meta-Analysis (PRISMA-NMA) statement for reporting (15).

### 2.1 Data sources and search strategy

We conducted a systematic search across four databases, including PubMed, Web of Science, Cochrane Controlled Trials Register, and Embase. During the search process, we employed a combination of medical subject headings and free-text terms, encompassing topics such as mind-body exercise, Tai Chi, Qigong, Wu Qin Xi, Ba Duan Jin, Yi Jing Jin, Liu Zi Jue, Yoga, Pilates, Dance for older adults, anxiety, depression, and randomized controlled trials. The search was limited to English language publications and covered the period from the inception of the databases until July 28, 2023. Additionally, we conducted a reference search of the included articles to identify studies that met our predefined criteria. The detailed search strategy is provided in the **Appendix**.

### 2.2 Selection and exclusion criteria

Inclusion criteria: The inclusion criteria for this study are as follows: (a) The baseline characteristics of the study population included old adults aged ≥60 years, regardless of gender, duration of illness, or source of cases. Additionally, participants should not have any diseases that could impact mind-body exercise. (b) The experimental group receives various types of mind-body exercises, including Tai Chi, Qigong (such as Ba Duan Jin, Yi Jin Jing, Liu Zi Jue, and Wu Qin Xi), yoga, Pilates, and dance. The control group, on the other hand, receives a waiting list, standard care, health education, or treatment methods different from the aforementioned interventions. (c) In the included scales, only select a scale that can measure either anxiety or depression as one of the outcome indicators. However, the selected scale must present baseline values and endpoint values, or the difference between the two. If multiple scales are used to test the same indicator in a study, we will choose the results obtained from the scale that is more suitable for the study population (old adults). These scales are specifically designed to assess symptoms in old adults, have high reliability and validity, and are widely used in older adults. To reduce the differences between different scales, standardized mean differences (SMD) should be used for representation. Most anxiety measurements use self-reported anxiety scales, the Hamilton Anxiety Rating Scale, or other valid specific scales. Depression, on the other hand, is measured using self-assessment scales such as the Beck Depression Inventory, Geriatric Depression Scale, Hamilton Depression Rating Scale, or other scales considered specific. Of course, some scales assess both indicators simultaneously, such as the Hospital Anxiety and Depression Scale (HADS). (d) All studies must be randomized controlled trials.

Exclusion criteria: The following criteria will be used to exclude studies: (1) duplicate publications, case reports, clinical guidelines, review articles, and non-randomized controlled trials; (2) interventions with unclear or mixed interventions combining Tai Chi with other exercises; (3) studies without analyzable data, such as studies that do not report values (i.e., mean, standard deviation, and sample size) necessary for calculating effect sizes; (4) participants with unclear age descriptions or <60 years old.

### 2.3 Data extraction and quality assessment

To eliminate duplicate search results, we utilized the EndNote X9 software. Subsequently, Subsequently, two reviewers independently assessed the titles and abstracts of the articles to determine their eligibility for inclusion in the study. For studies that did not meet the inclusion criteria, no further review was conducted. The remaining studies that were not excluded underwent a full-text evaluation by the two reviewers (YD and XK). Any disagreements or ambiguities were resolved through discussion with a third reviewer (JY). The data from randomized controlled trials (RCTs) were independently extracted by two reviewers according to a standardized form. The extracted information included the first author’s name, publication year, sample size, gender ratio, age, type of mind-body exercise intervention, control group intervention, intervention duration, frequency, cycle, primary outcome measures, and measurement tools. In case of unclear or incomplete information in the studies, the first author was contacted for clarification.

The two reviewers (DY and KX) assessed the quality of the included RCTs using the Cochrane risk of bias tool (16) in the following domains: (1) random sequence generation, (2) allocation concealment, (3) blinding of participants and personnel, (4) blinding of outcome assessors, (5) incomplete outcome data, (6) selective reporting, and (7) other biases. Each domain was evaluated as having a high risk of bias, an uncertain risk of bias, or a low risk of bias. Disagreements were resolved through discussion between the two reviewers, and if no consensus was reached, a third reviewer was consulted for final decision-making.

### 2.4 Data synthesis and analysis

During the data analysis process, statistical analysis was performed using Stata 15 software. To address the differences in anxiety and depression assessment scales used in different studies, standardized mean differences (SMD) were used as the measure of effect size for summarizing the results. Considering that SMD computed using Cohen’s d in small-scale studies may introduce slight bias and overestimate the effect size, our study utilized Hedges’ g as a summary measure to control for this bias (17). Network meta-analysis (NMA) was employed to explore the comparative relationships between different mind-body exercise interventions. For each outcome, SMDs, mean values, and 95% confidence intervals were calculated, with P<0.05 indicating statistical significance. Heterogeneity was assessed using statistical tests, where P>0.10 and I^2^<50% indicated low heterogeneity and a common-effect model was used; while P<0.10 and I^2^>50% indicated high heterogeneity and a random-effects model was used, simultaneously, we employ grouping analysis and meta-regression methods to explore the latent variables that lead to heterogeneity, while the REML method is used for testing heterogeneity (18). A network plot was created to visually depict the connections between different mind-body exercise interventions. The net-meta command in Stata 15 was used in conjunction with frequency analysis methods to perform the network meta-analysis. To assess potential inconsistency between direct and indirect evidence, a global consistency test was conducted, and node splitting was used to determine local consistency. If the analysis yielded a P>0.05, indicating no significant differences between direct and indirect comparisons, a consistency model was used to analyze the effect sizes of multiple treatment comparisons. Otherwise, an inconsistency model was employed. In cases where closed loops were present in the comparative studies, a test for inconsistency in the loops was conducted, with a 95% confidence interval (CI) containing 0 indicating that no significant loop inconsistency (19, 20).

We utilize a joint analysis table to compare the results of different intervention measures based on network meta-analysis. Additionally, by examining the area under the cumulative probability curve, we can rank the interventions, with a higher SUCRA value indicating a more effective intervention (21). Furthermore, to assess publication bias, we employ a comparison-adjusted funnel plot to detect potential publication bias risks. If the effect sizes of the included studies exhibit a symmetrical distribution, it suggests minimal publication bias in the network meta-analysis (22, 23).

## 3 Results

### 3.1 Study inclusion and selection

Through systematic retrieval, we have collected a total of 42 randomized controlled trials (RCTs) from 4 databases, encompassing 2974 old adults, and provided a detailed description of the literature screening process. After excluding 325 duplicate articles, we conducted a preliminary review of titles and abstracts, excluding 681 irrelevant articles and 206 meta-analyses. Finally, 160 articles were included for full-text reading. After a comprehensive review, 108 articles were excluded, including 2 conference abstracts, 1 clinical guideline, 2 inappropriate intervention measures, 57 articles with unavailable data, 7 articles without full-text access, 8 review articles, and 41 articles with participants’ average age below 60 years. In the end, we included 42 published randomized controlled trials in this network meta-analysis to compare the effects of different types of mind-body exercise interventions with the control group on improving anxiety and depression in older adults.

### 3.2 The characteristics of studies

**Figure 1:**
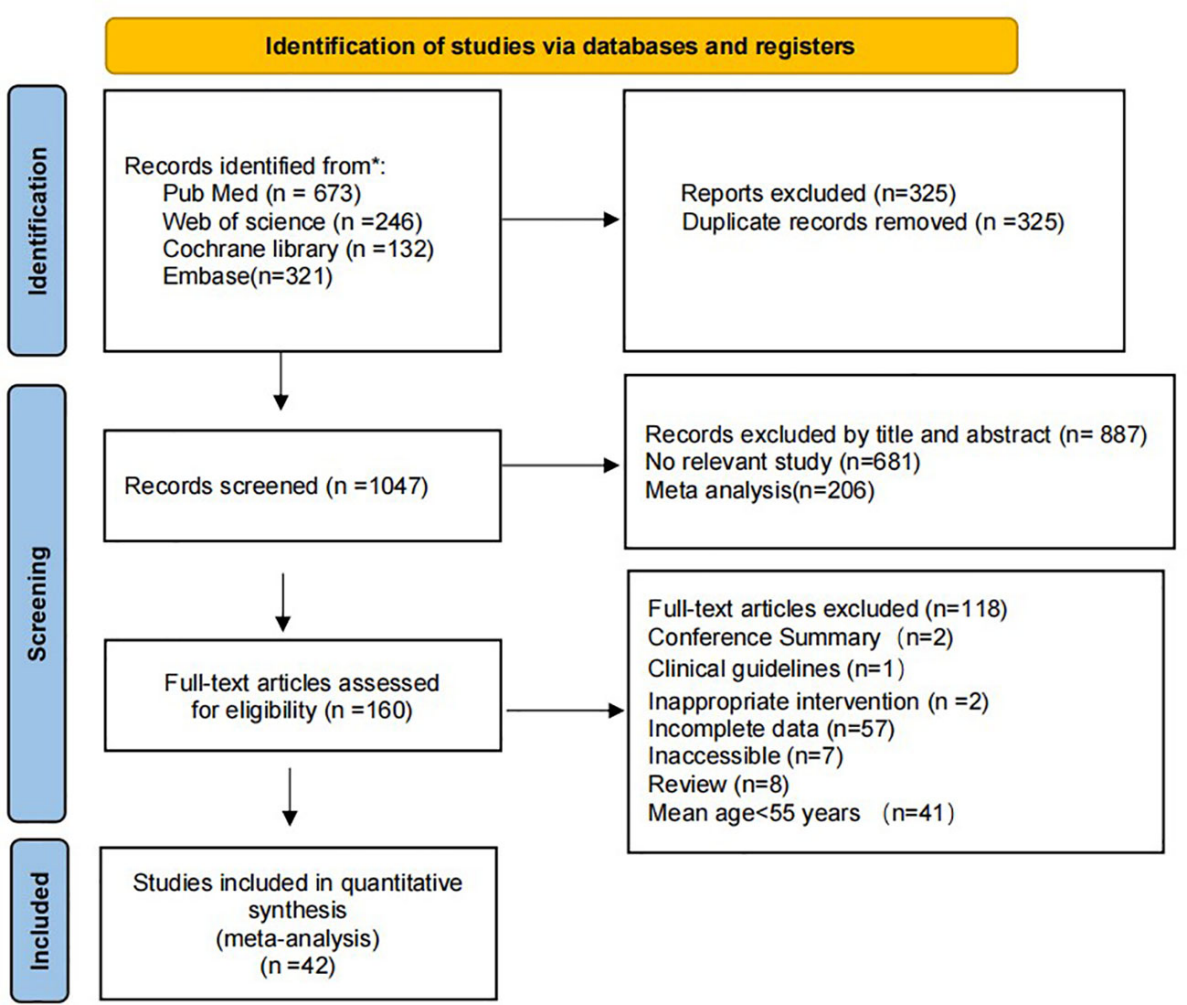
The process of slection of the eligible studies. * means reporting the number of records identified from each database or register searched (rather than the total number across all databases/registers).

This study covers 42 studies, including a total of 2974 old adults. All participants were randomly assigned to the experimental group and control group according to a predetermined protocol. The experimental group included five different intervention modalities (Tai Chi, Qigong, Yoga, Dance, and Pilates), including 23 studies involving Tai Chi, 8 involving Qigong, 5 involving Yoga, 6 involving Dance, and 1 involving Pilates. The duration of each intervention ranged from 20 to 90 minutes, and the frequency ranged from once a week to seven times a week. The control group received interventions such as routine daily activities, health education, physical exercise, and routine care. These studies are mainly distributed in China and the United States, with the publication years mainly concentrated between 2016 and 2022. Anxiety was evaluated using the Death anxiety scale, Self-rating Anxiety Scale, Hospital Anxiety and Depression Scale, and Hamilton Rating Scale of Anxiety, while depression was assessed using the Geriatric Depression Scale, Self-rating depression Scale, The 21-item Beck Depression Inventory−1A, Cornell Scale for Depression in Dementia, Center for epidemiologic studies depression scale, Hospital Anxiety and Depression Scale, Inventory of Depressive Symptomatology, Hamilton Rating Scale of Depression, Hamilton Rating Scale of Depression, and Cornell Medical Index. Within these 42 randomized controlled trials, the process of selecting eligible studies is shown in **Figure 1**, and the characteristics of the selected studies for this network meta-analysis are summarized in **Table 1**.

**Table 1:** Detailed characteristics of each of the included literature studies.

Study	Country	Sample size		Mean age years		Intervention		Frequency and period	Outcome measures	Outcome	Health status
		E	C	E	C	E	C				
Babaei Bonab S(2022 (24)	Iran	35	35	60.00-70.00		Tai Chi	routine daily activities	three 60-min sessions /12weeks	DAS	Anxiety	Health
Liu J (2018) (25)	China	30	30	60.90 ± 4.28	61.72 ± 3.54	Tai Chi	usual lifestyle	three 60-min sessions /24weeks	GDS	Depression	Mild Depression
Ge Y (2022) (26)	China	32	33	70.16 ± 5.40	72.91 ± 6.61	Tai Chi	routine daily activities	three 60-min sessions/8weeks	GDS	Depression	Health
Song J (2022) (27)	China	20	20	64.15 ± 8.56	64.15 ± 8.56	Tai Chi	health education	three 60-min sessions /12weeks	SAS	Anxiety	Health
									SDS	Depression	Health
Li Y (2019) (28)	China	163	163	63.61 ± 6.62	65.44 ± 5.79	Tai Chi	physical exercise	seven 60-min sessions /6 months	SAS	Anxiety	Mild Anxiety
									SDS	Depression	Mild Depression
Redwine LS (2019) (29)	USA	24	23	63.00 ± 9.00	67.00 ± 7.00	Tai Chi	usual care	Two 60-min sessions /16weeks	BDI	Depression	Mild depression
Lam L (2014) (30)	China	171	218	77.20 ± 6.30	78.30 ± 6.60	Tai Chi	physical exercise	three 30-min sessions /1year	CSDD	Depression	Health
Hsu CY (2016) (31)	China	30	30	80.70 ± 9.68	81.77 ± 6.32	Tai Chi	routine daily activities	three 40min sessions/26weeks	GDS	Depression	Health
Huang N (2019) (32)	China	36	38	81.90 ± 6.00	81.90 ± 6.10	Tai Chi	usual care	three 20 min sessions/10 months	GDS	Depression	Health
Chou KL (2004) (33)	China	7	7	72.60 ± 4.20		Tai Chi	usual care	three 45min sessions/3months	CES-D	Depression	Severe depression
Ma C (2018) (34)	China	79	79	70.24 ± 0.25	69.70 ± 10.84	Tai Chi	usual care	Three to five 90 sessions/6 months	CES-D	Depression	Health
Leung RW (2013) (35)	Australia	19	19	73.00 ± 8.00		Tai Chi	usual care	Five 30min sessions/12weeks	HADS	Anxiety	Health
										Depression	
Yeh GY (2020) (36)	American	52	29	68.60 ± 9.20	68.10 ± 6.70	Tai Chi	health education	Three 30min sessions/12weeks	CES-D	Depression	Health
Yıldırım P (2016) (37)	Turkey	30	30	62.90 ± 6.50	64.40± 7.50	Tai Chi	physical exercise	three1hour sessions/12weeks	GDS	Depression	Health
Solianik R (2021) (38)	Lithuania	15	15	≥60.00		Tai Chi	usual care	Two 60min sessions/10weeks	HADS	Anxiety	Health
										depression	
Irwin MR(2014) (39)	USA	48	25	66.30 ± 7.40	66.40 ± 7.70	Tai Chi	sleep seminar education	120min/week for 4 months	IDS-C	Depression	Health
Kilpatrick LA(2022) (40)	USA	21	19	67.10 ± 7.40	68.20 ± 5.40	Tai Chi	health education	One 60min sessions/12months	GDS	Depression	Mild Depression
Lavretsky H(2022) (41)	USA	89	89	69.20± 6.90	69.40± 6.20	Tai Chi	Health Education	60 minutes per week for 12 weeks	HAMA	Anxiety	Mild Anxiety
Chan AW(2017) (42)	China	24	24	75.40 ± 5.90	79.40 ± 8.50	Tai Chi	usual care	Two 60-minute for 3 months	MhI-18	Anxiety	Health
										Depression	
Lavretsky H(2011) (43)	Turkey	33	35	69.10 ± 7.00	70.0 ± 7.40	Tai Chi	Health Education	One 2hours sessions/10weeks	HADS	Anxiety	Mild Anxiety
										Depression	Severe depression
Yeh GY(2012) (44)	USA	8	8	68.00 ± 11.00	63.00± 11.00	Tai Chi	aerobic exercise	Two 1-hour sessions/12 weeks	POMS scale	Depression	Health
Brian Frye(2007) (45)	Netherlands	23	21	69.2 ± 9.26	69.2 ± 9.26	Tai Chi	memory enhancement training	one 40min sessions/12 weeks	CES-D	Depression	Mild Depression
Roswiyani R(2020) (46)	Indonesia	67	65	71.90 ± 8.57	74.31 ± 9.57	Qigong	routine daily activities	Two 90min sessions/8weeks	BDI	Depression	Mild depression
Tsang HW(2013) (47)	China	21	17	79.67 ± 6.55	80.65 ± 4.36	Qigong	usual care	three 45-min sessions/12 weeks	HRSD	Depression	Mild Depression
Lee P(2019) (48)	China	14	14	≥60.00		Qigong	cognitive training	twice per week/12 weeks	DASS-A	Depression	Health
Sun P(2022) (49)	China	30	30	62.03 ± 7.37	65.23 ± 6.29	Qigong	physical exercise	Seven 40min sessions/3weeks	HAMD	Depression	Mild Depression
Carcelén-Fraile MDC(2022) (50)	Spain	57	60	69.70 ± 6.15	69.75 ± 6.76	Qigong	routine daily activities.	Two 60-min sessions/12weeks	HADS	Anxiety	Mild Depression
										Depression	
Martínez N(2014) (51)	Spain	29	29	76.10 ± 8.10	72.50 ± 8.00	Qigong	usual care	two 90-min session/4-week	GDS	Depression	Health
Jing L(2018) (52)	China	39	40	75.25 ± 6.819	75.08 ± 5.264	Qigong	cognitive training	Two 1 to 1.5 hour/month for 3 months	GDS	Depression	Health
Zhang YX(2020) (53)	China	21	24	67.48 ± 5.05	67.63 ± 5.17	Qigong	usual care	One 30min session/12weeks	SAS	Anxiety	Health
Krause-Sorio B (2022) (54)	USA	11	11	61.45 ± 6.58	64.55 ± 6.41	Yoga	memory enhancement training	One 60min/session/12weeks	HAMA	Anxiety	Health
									BDI	Depression	Mild Depression
Kwok JYY (2019) (55)	China	71	67	63.70± 8.20	63.50 ± 9.30	Yoga	Stretching and Resistance Training Exercises	One 90-minute session/8 weeks	HADS	Anxiety	Health
										Depression	
Choi MJ (2018) (56)	China	33	30	77.60 ± 5.69	78.80 ± 5.83	Yoga	videotape	Four 30-40min session/12weeks	GDS	Depression	Mild Depression
Eyre HA (2016) (57)	Brazil	14	11	67.10 ± 9.50	67.80 ± 9.70	Yoga	memory enhancement training	One 60min session/12 weeks	GDS	Depression	Health
Noradechanunt C (2017) (10)	Australia	13	13	67.20 ± 8.30	65.20 ± 6.70	Tai Chi	physical exercise	Two 90min sessions/24weeks	CES-D	Depression	Health
		13	13	67.60 ± 4.90	65.20 ± 6.70	Yoga	home-based physical activity	Two 90min sessions/24week	CES-D	Depression	Health
Chang J (2021) (58)	China	62	47	76.56 ± 3.60	75.94 ± 3.61	Dance	normal life	three 30min sessions/18weeks	IDS-C	Depression	Health
Haboush A (2006) (59)	USA	9	9	69.38 ± 5.43	69.38 ± 5.43	Dance	waiting list	one 45min sessions/8weeks	GDS	Depression	Mild Depression
Hashimoto H(2015) (60)	Japan	15	14	67.90 ± 7.00	69.70 ± 4.00	Dance	normal life	One 60min sessions/12weeks	SDS	Depression	Health
Lee HJ(2017) (61)	USA	25	16	65.80 ± 7.20	65.70 ± 6.40	Dance	waiting-list	Two 60min sessions/8weeks	BDI	Depression	Mild depression
Mavrovouniotis FH(2008) (62)	Greece	76	35	67.62 ± 6.29	74.51 ± 6.78	Dance	usual care	One 60min sessions/10weeks.	SAS	Anxiety	Mild Anxiety
Ventura MI(2016) (63)	United State	8	7	71.80 ± 3.60	70.4 ± 5.50	Dance	usual activities	One 1.25hour sessions/4.5 months	GDS	Depression	Health
Curi VS(2018) (64)	Brazil	31	30	64.25± 0.14	63.75± 0.08	Pilates	usual care	Two 1hour sessions/16 weeks	GDS	Depression	Health

E, experiment group; C, control group; DAS, death anxiety scale; GDS, Geriatric Depression Scale; SAS, Self-rating Anxiety Scale; SDS, Self-rating depression Scale; BDI, Beck Depression Inventory; CSDD, Cornell Scale for Depression in Dementia; CES-D, Center for epidemiologic studies depression scale; Mental health inventory, MhI-18; HADS, Hospital Anxiety and Depression Scale; IDS-C, Inventory of Depressive Symptomatology; HAMA, Hamilton Rating Scale of Anxiety; HRSD, HAMD, Hamilton Rating Scale of Depression; CMI, Cornell Medical Index.

### 3.3 Quality assessment

**Figure 2:**
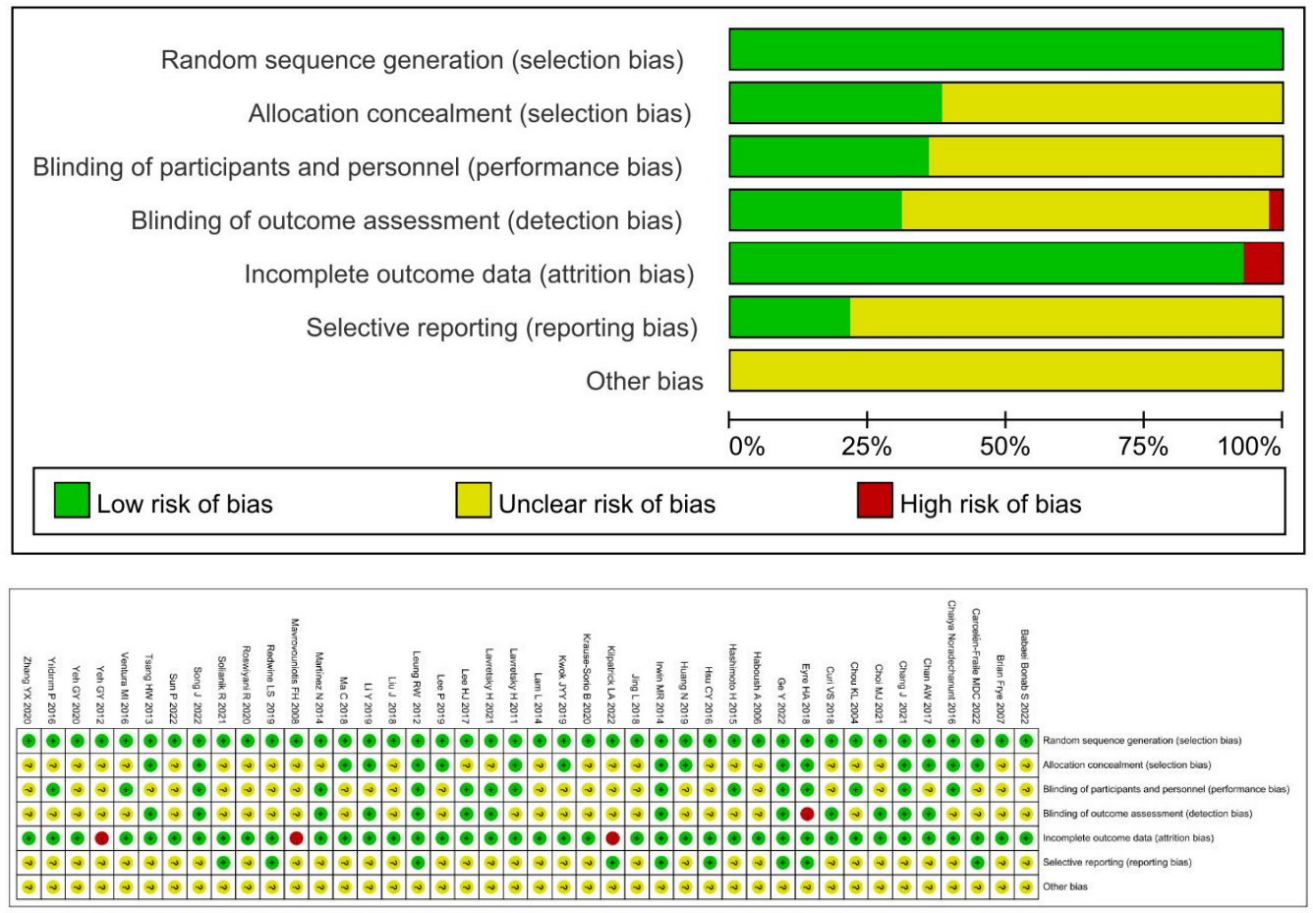
Quality assessment of included studies.

The quality assessment of the selected studies was conducted using the Cochrane risk of bias tool, and the assessment results are shown in **Figure 2**. 41 studies were two-arm studies, with one being a three-arm study, all involving descriptions of randomization. Among them, 27 studies described in detail the methods of generating random sequences, while only 8 studies mentioned specific allocation concealment methods. Due to the nature of physical exercise interventions, most experiments did not use blinding, with only a few experiments using a single-blinding design. The majority of experiments reported in detail the data missing during the experimental process, with 2 studies not specifying the specific reasons for participant loss during the experiment, thus being judged as high risk of bias. All studies did not report other biases.

### 3.4 Pairwise meta-analysis

Pairwise meta-analyses showed that the intervention group significantly reduced anxiety (SMD: -0.87, 95% CI: -1.43, -0.31, p<0.05) and depression (SMD: -0.52, 95% CI: -0.71, -0.34, p<0.05) scores compared to the control group. In addition, our findings also showed a high degree of heterogeneity in anxiety (I^2^ = 95%) and depression (I^2^ = 80%) after the meta-merger. For detailed results of the meta-analysis, please refer to the **Appendix**. To further explore the sources of heterogeneity, we conducted subgroup and meta-regression analyses based on moderating variables.

The moderating variables were categorized into 3 main areas. The first is the population characteristics moderating variable, which mainly includes the age and health status of the subjects. The second is the interventions, mainly including the experimental group interventions and control group interventions. The third is the evaluation scale. Subgroup analyses in this study showed that subgroup analyses based on moderator variables did not significantly reduce the heterogeneity of anxiety and depression outcome indicators. For anxiety indicators, in the subgroup with age >70 years, mind-body exercise had no significant effect. In the experimental group, different intervention methods also showed that qigong had no significant effect. As for the indicators of depression, the subgroup analysis of different intervention methods in the experimental group showed that dance had no significant effect, while in the control group, different intervention measures of physical exercise showed that mind-body exercise had no significant effect on depression. Among the different subgroups using different evaluation scales, BDI showed that mind-body exercise also had no significant effect on depression. Meta-regression analyses showed that differences in the evaluation scales were a significant contributor to heterogeneity in the anxiety outcome indicators (P=0.035), while the remaining moderator variables did not affect heterogeneity; please see **Appendix Tables S2**, **S3** for detailed subgroup analyses, and **Appendix Tables S4**, **S5** for meta-regression results.

### 3.5 Network meta-analysis

**Figure 3:**
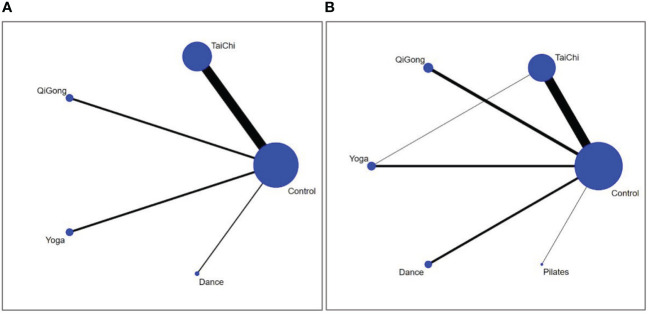
The network structure of the analysed treatment comparisons for the outcome of anxiety **(A)** and depression **(B)**.

In the included 42 studies, a network diagram (**Figure 3**) was established between different forms of mind-body exercises in the intervention of anxiety and depression in older adults. The lines between the points in the diagram represent studies with direct comparisons, while points without lines indicate randomized controlled trials that have not been directly compared. The thicker the line between two points in the network diagram, the more studies have compared these two intervention measures. A consistency test was conducted for the anxiety and depression outcome indicators included in the studies. The results show that the PSRF parameter values are all greater than 0.05, close to 1.00, indicating good convergence of the model. Therefore, a network meta-analysis was conducted under the assumption of consistency.

**Figure 4:**
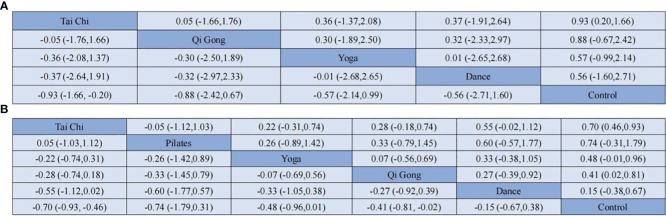
Results of the network meta-analysis (SMD vs 95% CI) of the effects different mind-body exercise on anxiety **(A)** and depression **(B)** in the adults.

**Figure 5:**
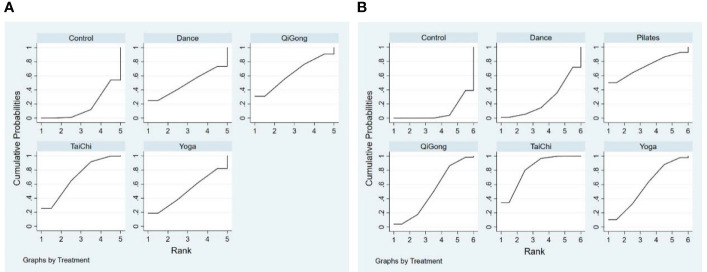
Anxiety **(A, B)** depression rank probability.

Network meta-analyses covered changes in anxiety for 4 different types of mind-body exercise interventions versus a control group (**Figure 3A**). Since there is no direct and indirect comparison in the network plot, we used the consistency model to compare different interventions. The specific results are shown in **Figure 4A**, compared with the control group, Tai Chi (SMD: -0.93, 95% CI: -1.66, -0.20), Qigong (SMD: -0.88, 95%CI: -2.42,0.67), Yoga (SMD: -0.57, 95%CI: -2.14, 0.99), Dance (SMD: -0.56,95% CI: -2.71, 1.60) all showed better effects. In the probability ranking table in **Figure 5A**, Tai Chi exercise is considered to be the most likely intervention for anxiety in older adults, with a probability of 70.4%, followed by Qigong (63.4%). Therefore, according to this ranking, the intervention effects of the four mind-body exercises on anxiety in older adults are as follows: Tai Chi > Qigong > Yoga > Dance > Control group.

The depression network diagram shown in **Figure 3B**), was compared between the 5 different mind-body exercise interventions and the control group. Considering the presence of both direct and indirect comparisons in the network diagram, we first performed an inconsistency test. The results showed good convergence. In addition, considering that a triangle loop was formed by a multi-arm trial in this study, the consistency of the I^2^ quadratic loop was tested if they were inconsistent. The 95% confidence interval (CI) of the closed loop was 0, indicating a significant inconsistency. Therefore, a consistency model was adopted to compare different interventions. The results in **Figure 4B** showed that in comparison with the control group, Tai Chi (SMD: -0.70, 95% CI: -0.93, -0.46), Pilates (SMD: -0.74, 95% CI: -1.79, 0.31), Yoga (SMD: -0.48, 95% CI: -0.96, -0.01), Qigong (SMD: -0.41, 95% CI: -0.81, -0.02), and Dance (SMD: -0.15, 95% CI: -0.67, 0.38) showed better efficacy. In the probability ranking table in **Figure 5B**, Tai Chi was considered to be the most likely best intervention for old adults with depression with a probability of 83.7%, followed by Pilates (73.9%). Therefore, according to this ranking, the intervention effects of the five mind-body exercises on depression in older adults are as follows: Tai Chi > Pilates > Yoga > Qigong > Dance > control group.

### 3.6 Publication bias

**Figure 6:**
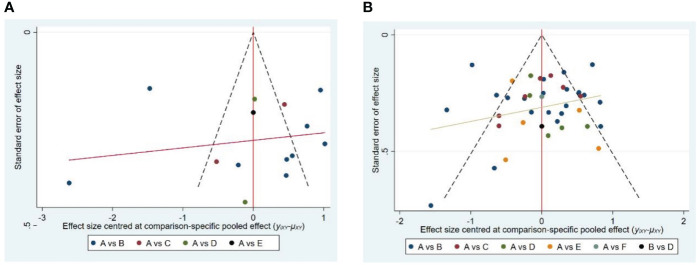
Publication bias of four tyles of mind-body exercise on anxiety **(A)** and depression **(B)**. In the picture **(A)**, A is control group, B is Tai Chi, C is Qigong, D is Yoga, and E is Dance. In picture **(B)**, A is control group, B is Tai Chi, C is Qigong, D is Yoga, E is Dance, and F is Pilates.

In the final inclusion of the 42 studies, 13 studies provided detailed descriptions of the intervention effects of different types of mind-body exercises on anxiety in older adults, while the other 38 studies reported intervention effects on depression. These studies all achieved a moderate to high level of quality assessment. In addition, we used anxiety and depression as outcome indicators and generated a funnel plot using STATA software version 15. The results showed that the funnel plot of depression (**Figure 6B**) presented a basic stacking pattern, indicating no apparent publication bias for the depression indicator. However, the funnel plot of anxiety (**Figure 6A**) exhibited some degree of asymmetry, which may be related to the relatively little literature on the included types of mind-body exercises and the relatively insufficient overall sample size. Therefore, there is a possibility of publication bias.

### 3.7 Sensitivity analysis

By excluding individual studies, sensitivity analysis demonstrated that there was no significant change in the statistical significance of all primary or secondary outcomes. This further validates the robustness of the results and the heterogeneity of the study.

## 4 Discussion

Mental health issues are increasingly gaining attention in the old adult population, with anxiety and depression, although common, often going unnoticed. This study employed a network meta-analysis approach, incorporating 42 articles with a total of 2,000 participants. Among these articles, 13 focused on anxiety, while 38 addressed Depression. We systematically explored mind-body exercises, including Tai Chi, Qigong, yoga, Dance, and Pilates, among various physical exercise modalities, by conducting direct and indirect comparative analyses of their interventions on anxiety and depression in older adults. In Pairwise meta-analysis, it is evident that mind-body exercises have significant effects on anxiety (SMD: -0.84, 95% CI: -1.37, -0.31, p<0.05, I^2 =^ 94%) and depression (SMD: -0.52, 95% CI: -0.71, -0.34, p<0.05, I^2 =^ 80%) among older adults. In the network meta-analysis, Tai Chi ranked highest in terms of SUCRA values for anxiety, followed by Qigong, yoga, Dance, and the control group. Similarly, for depression, Tai Chi demonstrated the most favorable outcomes, followed by Pilates, yoga, Qigong, Dance, and the control group.

A mind-body exercise is a comprehensive form of health practice, including Tai Chi, Qigong, yoga, dance, and Pilates, among others. It can alleviate physical and mental tension, improve mental health, and establish a healthier lifestyle to reduce anxiety and depression. It can also stimulate the synthesis of tryptophan hydroxylase, thereby promoting the production of serotonin (65). In addition, mind-body exercise can activate the endocannabinoid system, alter the function of the hypothalamic-pituitary-adrenal axis, increase levels of norepinephrine, decrease the expression of glucocorticoid receptors, and promote the regeneration of hippocampal neurons (66, 67). Interestingly, this study demonstrates that physical and mental exercise has a significant intervention effect on anxiety and depression in older adults. However, traditional meta-analyses have shown high levels of statistical heterogeneity. By including population characteristics (age and health status) as well as the classification of the experimental and control groups, and using different scales as moderating variables, it was found that the use of different scales is the cause of heterogeneity in anxiety indicators. This difference may be due to the variations in the assessment of anxiety indicators by different measurement scales, including question settings and scoring criteria, which leads to heterogeneity in the results. However, we cannot rule out other potential influencing factors, such as cultural and racial differences among the included population, or other variables that have not been considered.

Tai Chi, a traditional Chinese fitness practice, is well-known for its unique exercise approach, characterized by slow, fluid movements, deep breathing, and elements of meditation (68). These distinctive features contribute to lowering stress levels, reducing tension, and enhancing relaxation in the body, thus positively affecting mental well-being (69). Our research findings reveal that Tai Chi ranks highly in SUCRA values for addressing anxiety and depression in older adults, indicating its effectiveness as one of the primary methods. This is attributed to Tai Chi’s capacity to heighten bodily awareness and relaxation, which aids in mitigating tension and anxiety, ultimately raising levels of psychological well-being. Tai Chi underscores meditation and the connection between body and mind, facilitating superior emotional management (70). During Tai Chi practice, the emphasis on fluid body postures enhances concentration and self-harmony, critical factors in alleviating anxiety and depression (71). The deep breathing exercises in Tai Chi play a vital role in managing anxiety by stabilizing the autonomic nervous system, transitioning the body from a state of stress to one of calm, and reducing tension and anxiety, thereby enabling individuals to respond to stressors with greater composure (24). Additionally, Tai Chi’s meditation components help individuals focus, enhance self-awareness, and reduce negative thinking. This meditation practice strengthens emotional regulation, enabling better coping with depressive emotions (72).

Our study also compared other forms of mind-body exercises, including Qigong, Yoga, and Dance, to explore their effects on anxiety and depression intervention. Despite falling within the category of mind-body exercises, these interventions exhibit differences in their methods and practices, potentially resulting in varying outcomes in alleviating anxiety and depression. Qigong emphasizes the regulation of breath and the balance of internal energy. Deep breathing and specific movements (73) have shown some effectiveness in addressing anxiety and depression in older adults. Research results indicate that Qigong practice can significantly reduce anxiety and depression levels in older adults (74). However, what is even more crucial is Qigong’s focus on cultivating emotional management and self-regulation. By enhancing emotional stability, older adults can more effectively cope with anxious emotions, thereby reducing their severity (74). This improvement in emotional management plays a pivotal role in the treatment of anxiety and depression in older adults.

Yoga focuses on the practice of asanas (physical postures) and breath control techniques, which can lead to various physiological and psychological effects, thereby influencing the outcomes of anxiety interventions (75). The meditation and body posture exercises in yoga improve emotional stability and psychological well-being in older adults (76). A study has shown that yoga practice can significantly improve depression in family caregivers (77). However, more importantly, yoga emphasizes cultivating emotional management and self-awareness. By enhancing emotional stability, older adults are better able to cope with anxiety and depressive emotions, thereby reducing their severity (78). Improving emotional management skills is crucial for the psychological well-being of older adults.

Although Pilates primarily focuses on physical training, it still incorporates elements of deep breathing and body awareness in its practice (79). Research findings suggest that Pilates ranks high in interventions for reducing depression in older adults, which may be attributed to its emphasis on the connection between the body and emotions, thus aiding in improving emotional management and alleviating depression (80). However, it is important to note that this study only included one research article on Pilates, thus potential biases exist. Further research is needed to gain a more comprehensive understanding of the mechanisms of Pilates and its relationship with emotional management.

In addition, two meta-analytic studies have shown that Dance interventions do not have a significant effect on anxiety and depression (81, 82), which is consistent with our research findings. Dance emphasizes the body’s dynamism and the sense of rhythm in music, which may make it challenging for many older adults to adapt to its specific rhythms and demands (83). Apart from differences in intervention methods, the quality and quantity of research may also influence the relative effectiveness of these mind-body exercises. For example, some studies may have a small sample size or inadequate control over individual differences, all of which can impact the reliability and generalizability of the research.

Furthermore, it is imperative to consider the individual differences and intervention preferences of older adults, as they can significantly impact the effectiveness of mind-body exercise. Each older adult possesses unique physical conditions, health needs, and varying levels of acceptance toward different types of exercise modalities. Consequently, a particular mind-body exercise may prove more efficacious for certain individuals, while being less suitable for others, thereby elucidating the observed variations in outcomes. For instance, some older adults may find it easier to embrace the slow and gentle movements of Tai Chi, while being more receptive to the breath-regulation techniques of Qigong, thereby manifesting superior outcomes in anxiety and depression interventions. Conversely, there may be individuals who lean towards the mind-body balance offered by yoga or the muscle conditioning provided by Pilates, consequently exhibiting enhanced results in terms of psychological well-being. These factors of individual differences and intervention preferences necessitate further exploration in future research endeavors, to formulate personalized and precise mental health intervention plans.

There are several limitations to our study. First, we observed significant heterogeneity in the traditional meta-analysis phase, and although we used subgroup analysis and meta-regression to explore the sources of heterogeneity, we were not able to pinpoint the sources of heterogeneity, and future research could validate the results of this study by expanding the study sample size and using experimental studies with different interventions as well as different evaluation scales. Second, only one Pilates study was included in this study, which may also introduce bias in the findings. It is recommended that future studies expand the search and increase the number of study interventions to further validate the results of this study.

## 5 Conclusion

The network meta-analysis results of this study show that mind-body exercises have significant positive effects on improving anxiety and depression in older adults. Among the five different types of mind-body exercises included in the research, Tai Chi and Qigong showed better improvement in reducing anxiety. For older patients who wish to improve depression, we recommend considering Tai Chi and Pilates as treatment options. However, to scientifically select suitable exercise programs, individual differences of patients need to be fully considered. Therefore, this study provides evidence and references for clinical doctors regarding non-pharmacological treatment options for improving anxiety and depression in older adults.

## Data availability statement

The original contributions presented in the study are included in the article/**Supplementary Material**. Further inquiries can be directed to the corresponding author.

## Author contributions

YD: Writing – original draft, Validation, Software, Methodology, Data curation. JY: Writing – review & editing, Supervision, Formal analysis. XK: Writing – original draft, Validation, Software, Methodology, Data curation. RZ: Writing – review & editing, Methodology, Formal analysis. LC: Writing – original draft, Software, Methodology, Data curation. XZ: Writing – review & editing, Methodology, Supervision, Formal analysis.
