# Supplementary material for: The effects of mind-body exercise on anxiety and depression in older adults: a systematic review and network meta-analysis
Source: Front Psychiatry. 2024 Feb 7;15:1305295. doi: 10.3389/fpsyt.2024.1305295 (PMC10879425; doi:10.3389/fpsyt.2024.1305295)
Supplement: Supplementary file 1 [file DataSheet_1.docx]

Supplementary Material

Table S1 Search strategy in PubMed

| **Step** | **Search strategy** |
| --- | --- |
| #1 | (Aged[MeSH Terms]) OR (elderly[Title/Abstract]) |
| #2 | (((((((((((((((((((((((((((((((((((((Dance[MeSH Terms]) OR (Dance, Modern[Title/Abstract])) OR (Modern Dance[Title/Abstract])) OR (Dancing, Line[Title/Abstract])) OR (Line Dancing[Title/Abstract])) OR (Tap Dance[Title/Abstract])) OR (Dance, Tap[Title/Abstract])) OR (Ballet[Title/Abstract])) OR (Dance, Jazz[Title/Abstract])) OR (Jazz Dance[Title/Abstract])) OR (Dance, Square[Title/Abstract])) OR (Square Dance[Title/Abstract])) OR (Hip-Hop Danc[Title/Abstract])) OR (Hip Hop Dance[Title/Abstract])) OR (Dance, Hip-Hop[Title/Abstract])) OR (Dancing, Salsa[Title/Abstract])) OR (Salsa Dancing[Title/Abstract])) OR (Yoga [Title/Abstract])) OR (Tai Ji[MeSH Terms])) OR (Tai-ji[Title/Abstract])) OR (Tai Chi[Title/Abstract])) OR (Chi, Tai[Title/Abstract])) OR (Tai Ji Quan[Title/Abstract])) OR (Ji Quan, Tai[Title/Abstract])) OR (Quan, Tai Ji[Title/Abstract])) OR (Taiji[Title/Abstract])) OR (Taijiquan[Title/Abstract])) OR (T'ai Chi[Title/Abstract])) OR (Tai Chi Chuan[Title/Abstract])) OR (qigong[MeSH Terms])) OR (qi gong[Title/Abstract])) OR (Liu Zi Jue[Title/Abstract])) OR (Wu Qin Xi[Title/Abstract])) OR (Six Healing Sounds[Title/Abstract])) OR (Ba Duan Jin[Title/Abstract])) OR (Traditional Chinese exercise[Title/Abstract])) OR (Traditional Chinese medicine exercise therapy[Title/Abstract])) OR (Yi Jin Jing[Title/Abstract]) |
| #3 | ((((((((((((((Anxiety[MeSH Terms]) OR (Angst[Title/Abstract])) OR ("Social Anxiety"[Title/Abstract])) OR ("Anxieties, Social"[Title/Abstract])) OR ("Anxiety, Social"[Title/Abstract])) OR ("Social Anxieties"[Title/Abstract])) OR (Hypervigilance[Title/Abstract])) OR (Nervousness[Title/Abstract])) OR (Anxiousness[Title/Abstract])) OR (Depression[MeSH Terms])) OR ("Depressive Symptoms"[Title/Abstract])) OR ("Depressive Symptom"[Title/Abstract])) OR ("Symptom, Depressive"[Title/Abstract])) OR ("Emotional Depression"[Title/Abstract])) OR ("Depression, Emotional"[Title/Abstract]) |
| #4 | (((randomized controlled trial[Publication Type]) OR (controlled clinical trial[Publication Type])) OR (randomized[Title/Abstract])) |
| #5 | #1 AND #2 AND #3 AND#4 |

Note: we employed a combination of medical subject headings and free-text terms, encompassing topics such as mind-body exercise, Tai Chi, Qigong, Wu Qin Xi, Ba Duan Jin, Yi Jing Jin, Liu Zi Jue, Yoga, Pilates, Dance for older adults, anxiety, depression, and randomized controlled trials.


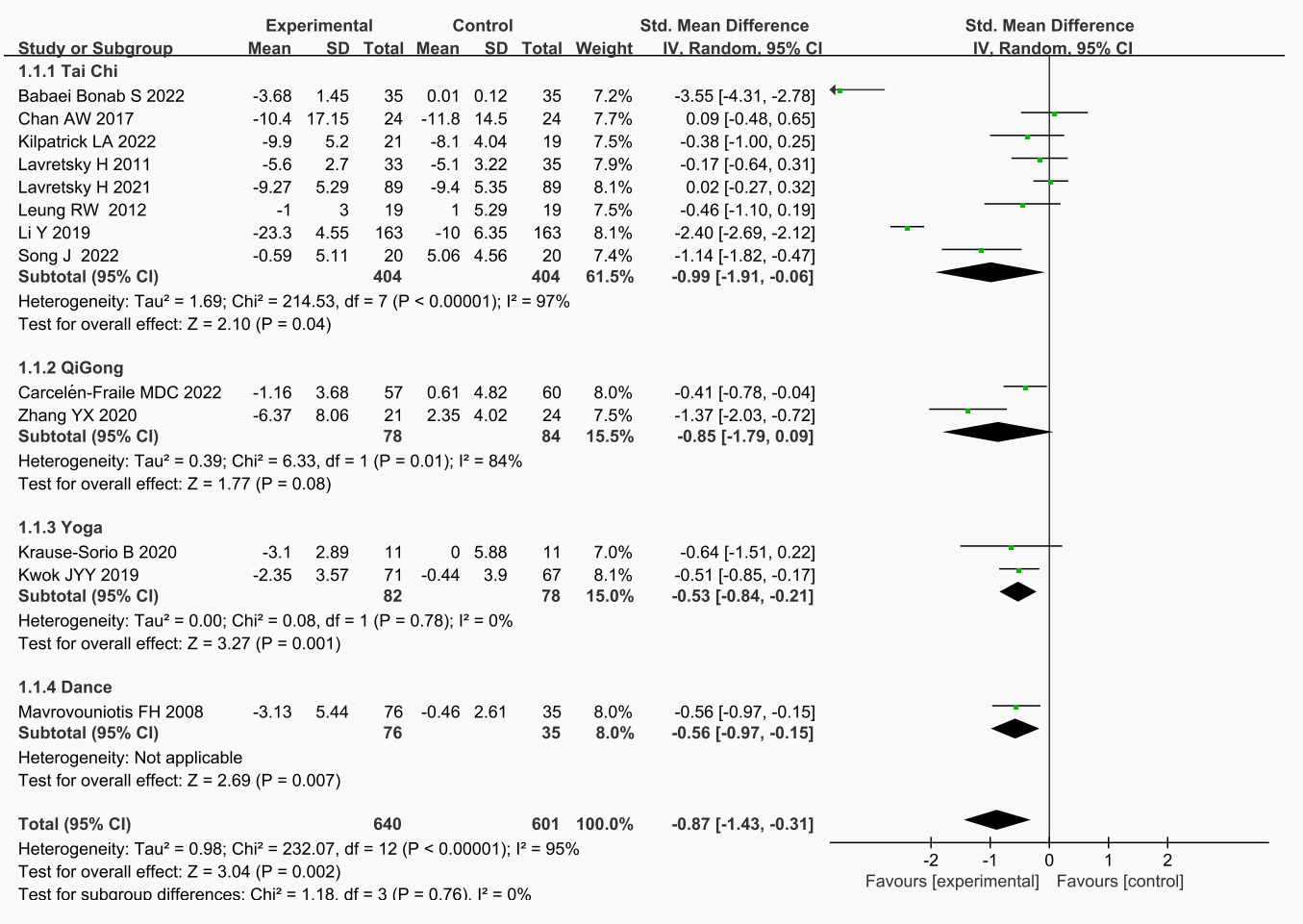


Figure S1 | Pairwise meta-analysis of mind-body exercise on anxiety


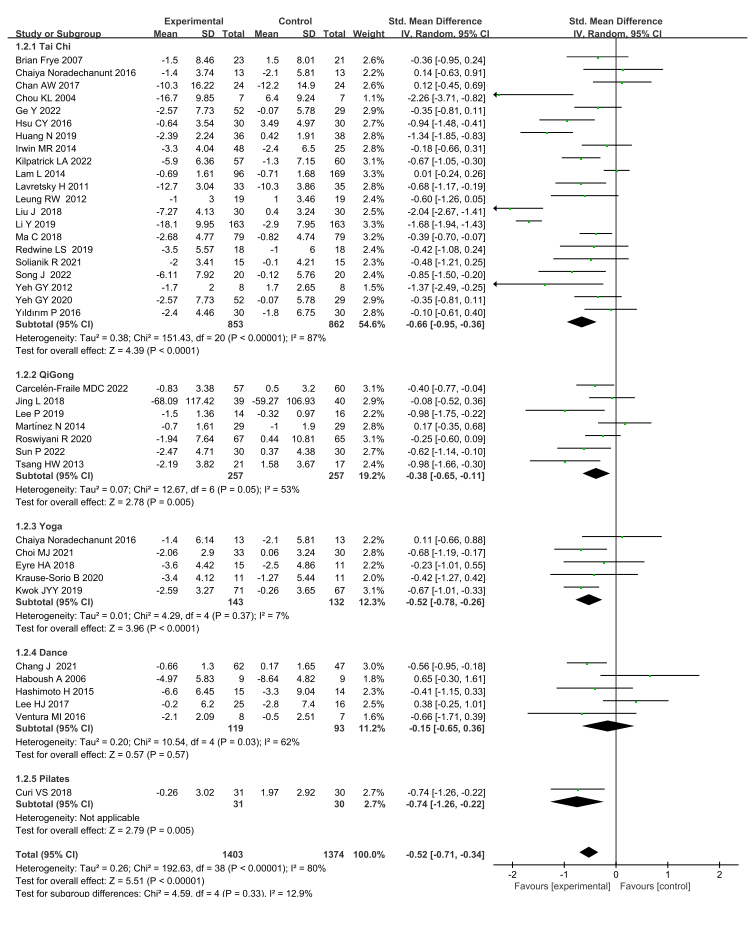


Figure S2 | Pairwise meta-analysis of mind-body exercise on depression


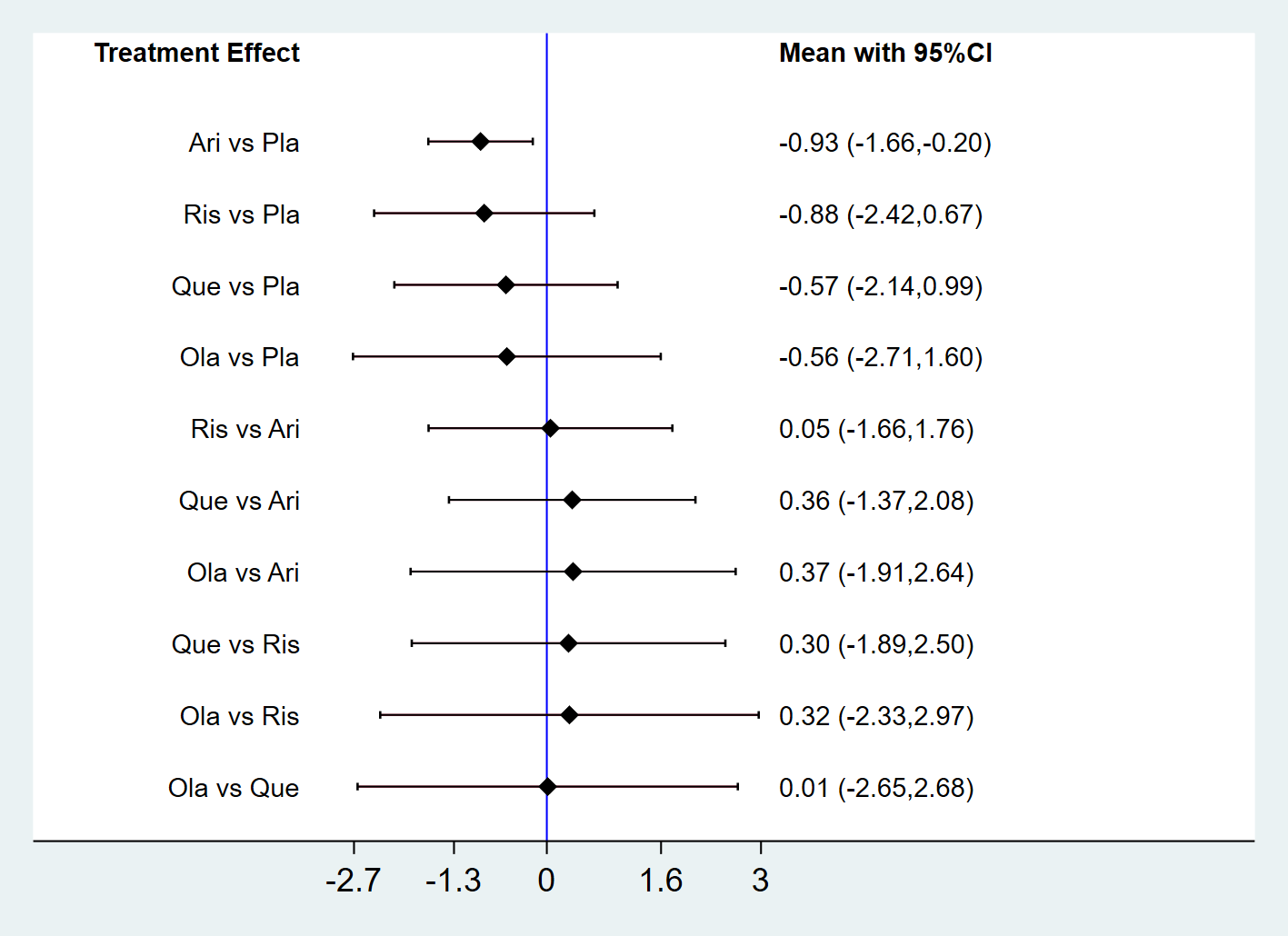


Figure S3 Interval plot of network meta-analysis for anxiety in the old adults


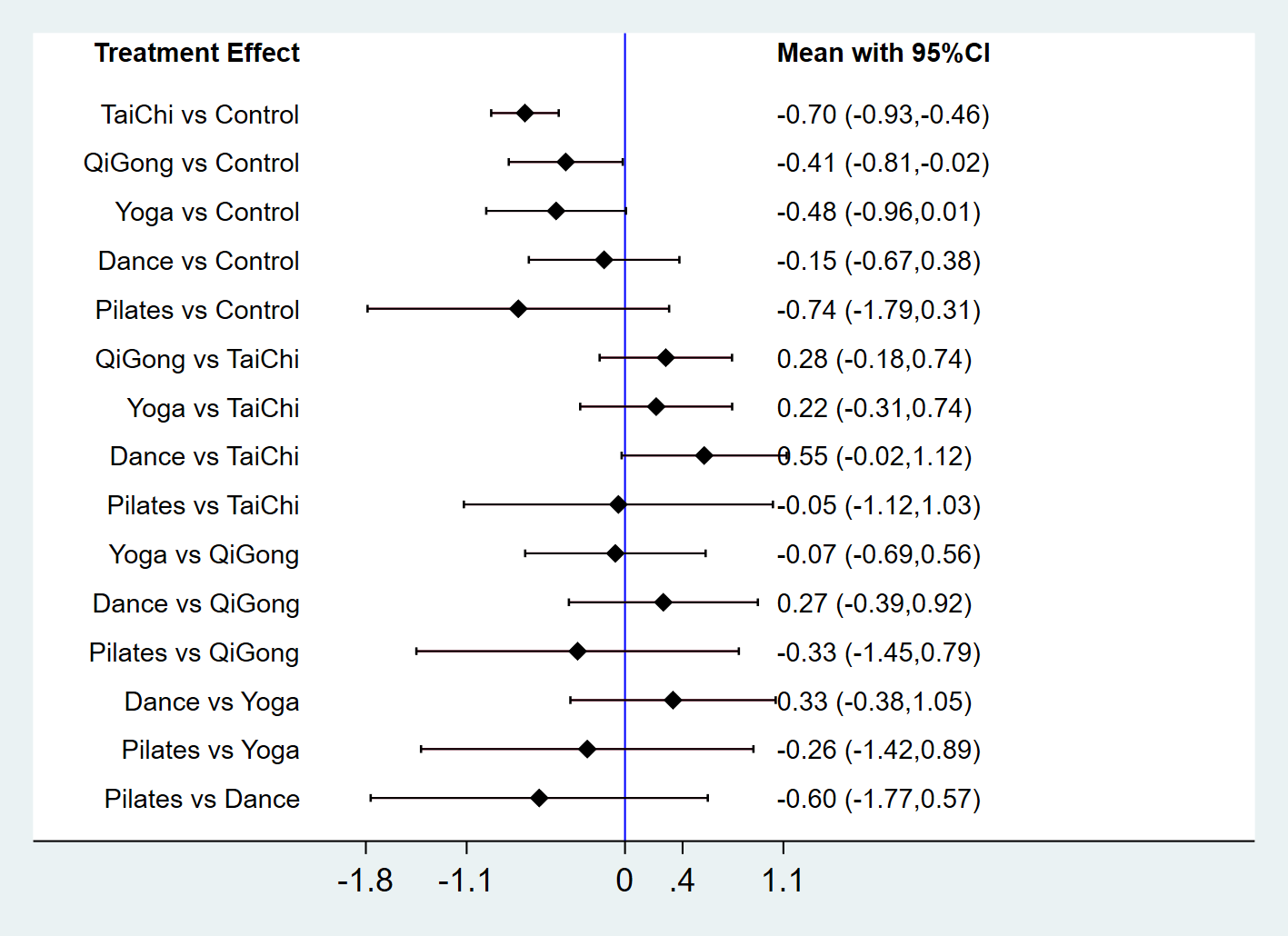


Figure S4 Interval plot of network meta-analysis for depression in the old adults


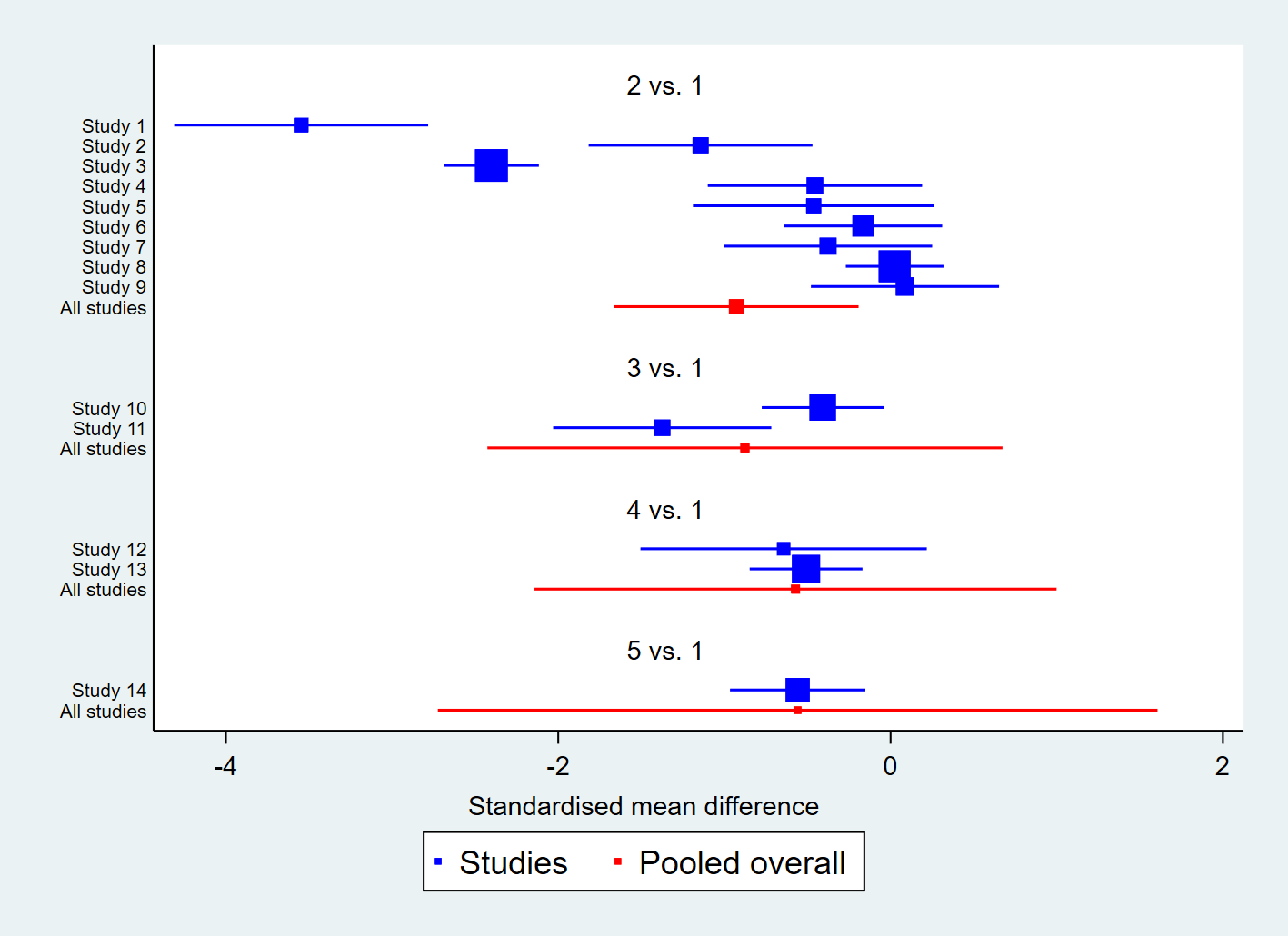


Figure S5 Forest map of the effect of mind-body exercise on anxiety of the old adults


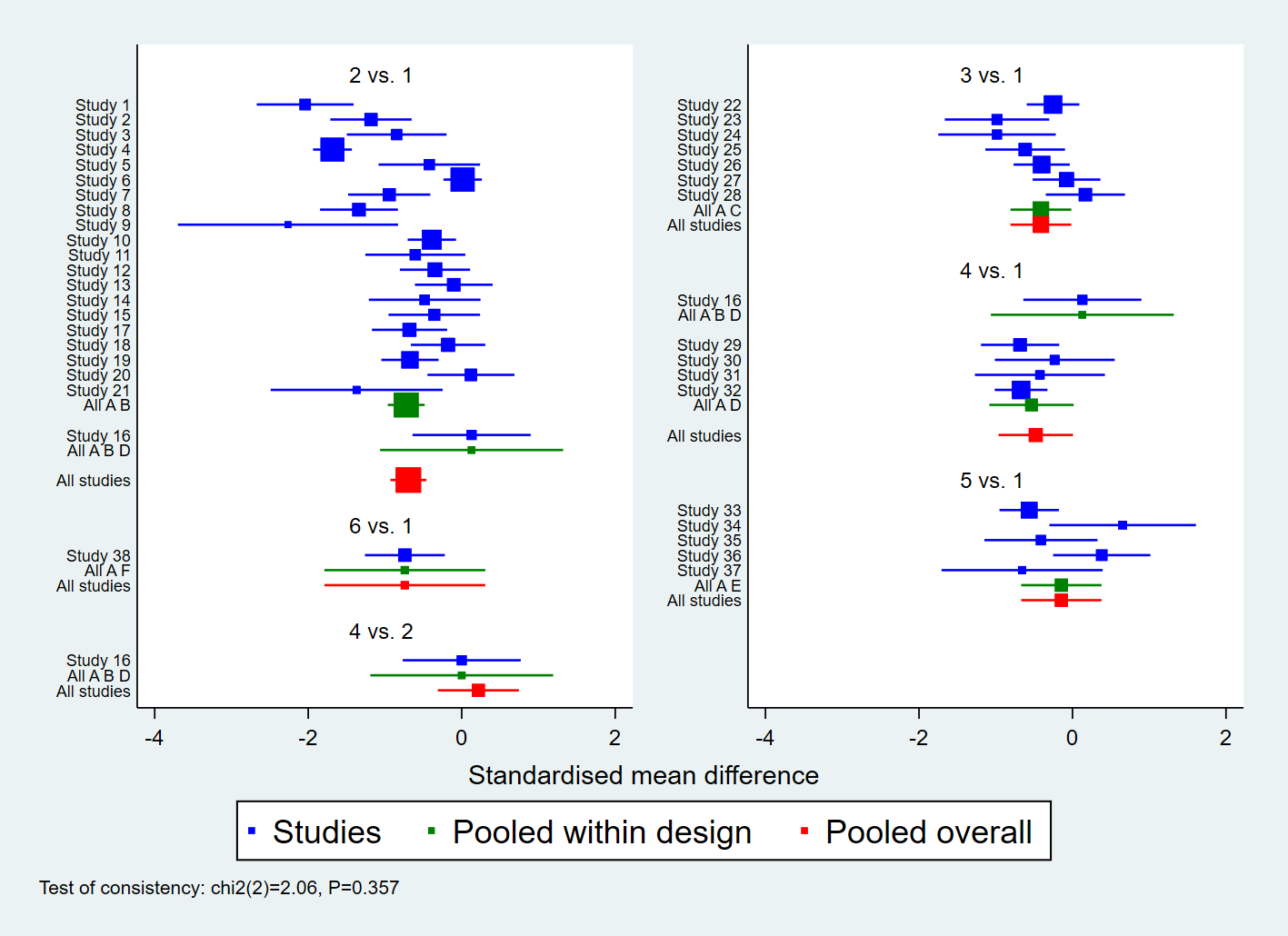


Figure S6 Forest map of the effect of mind-body exercise on depression of the old adults


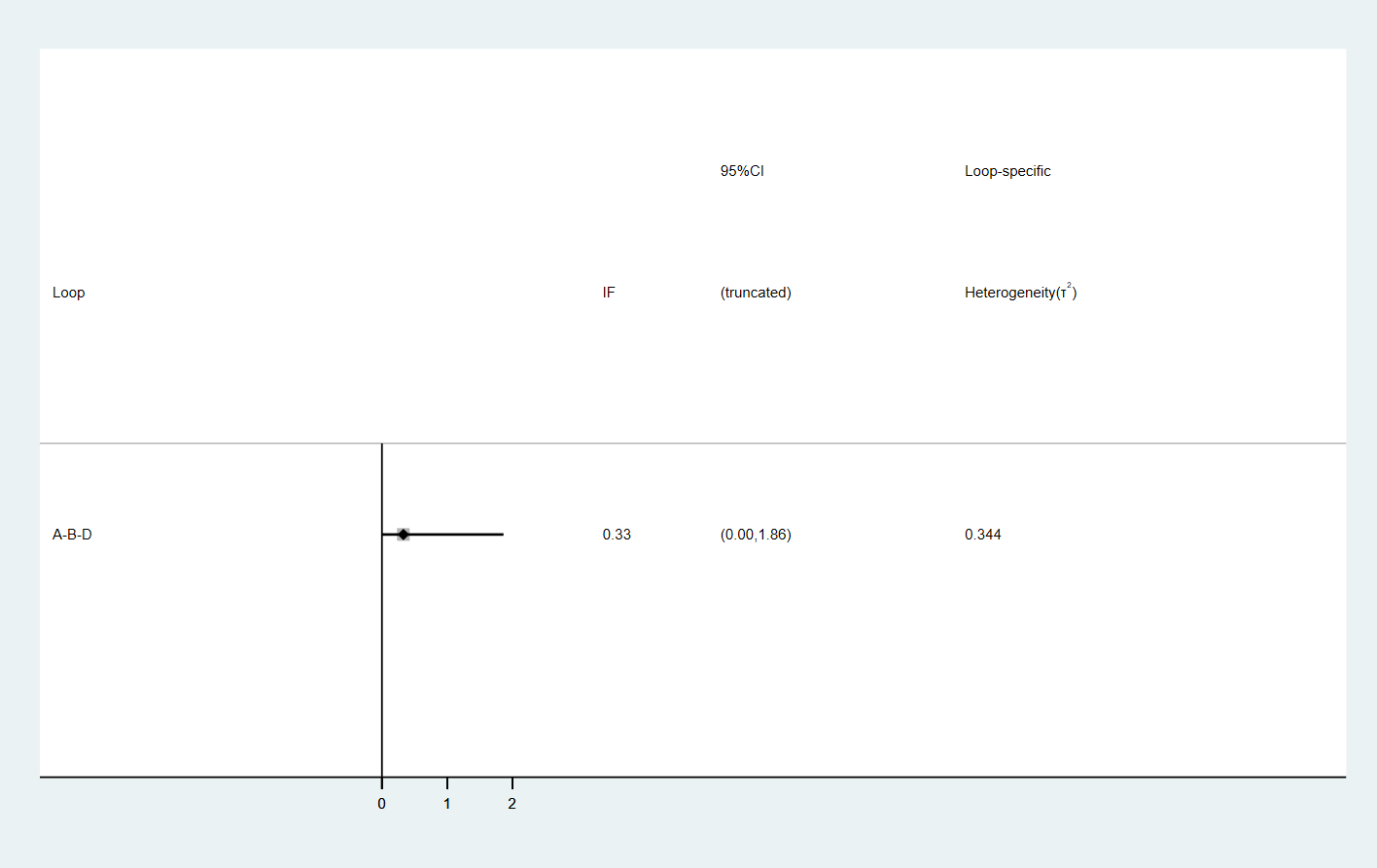


Figure S7 The loop inconsistency plot, A is control, B is Tai Chi, D is Yoga


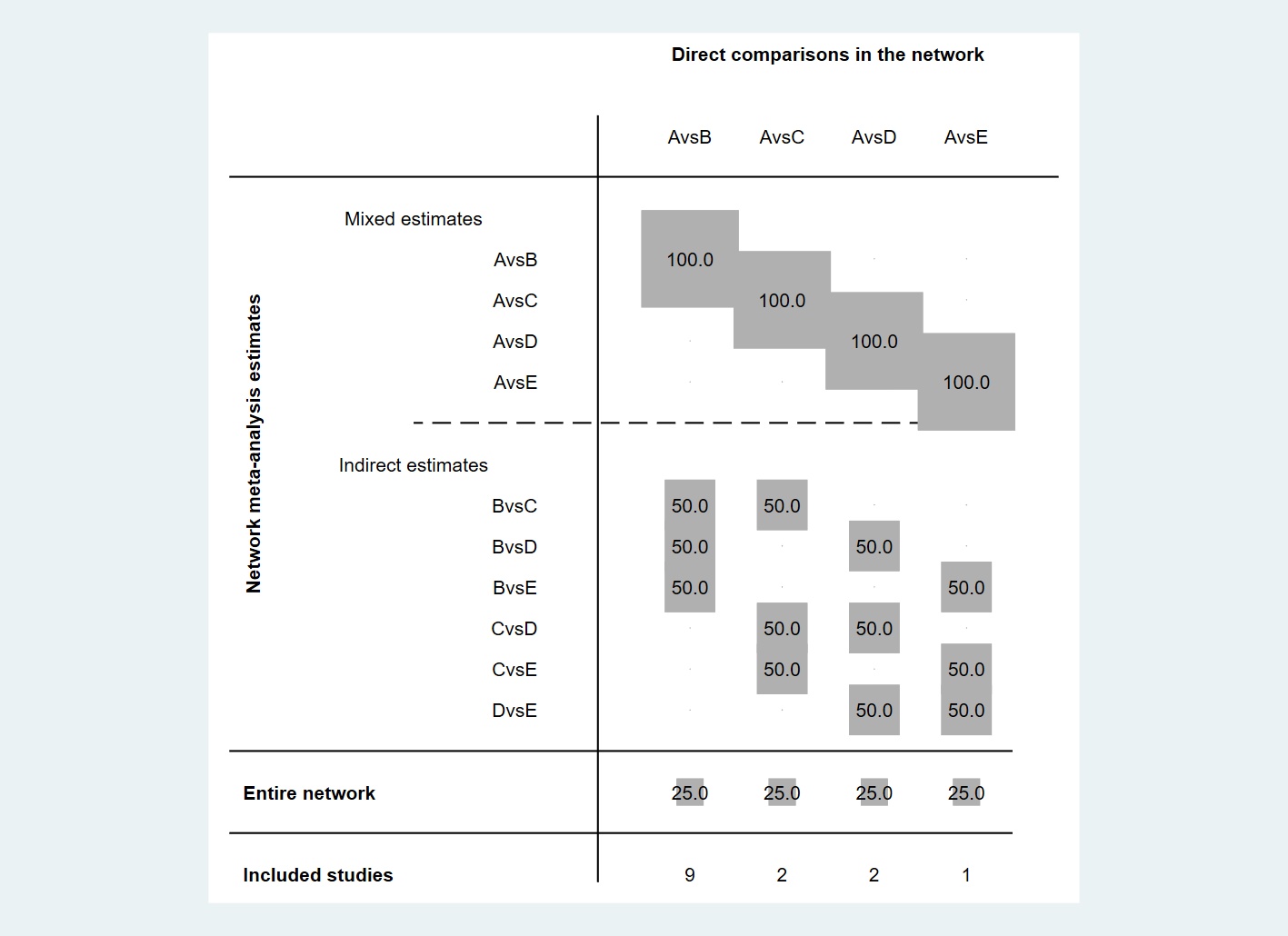
Figure S8 The Contribution of Anxiety Graph of the old adults
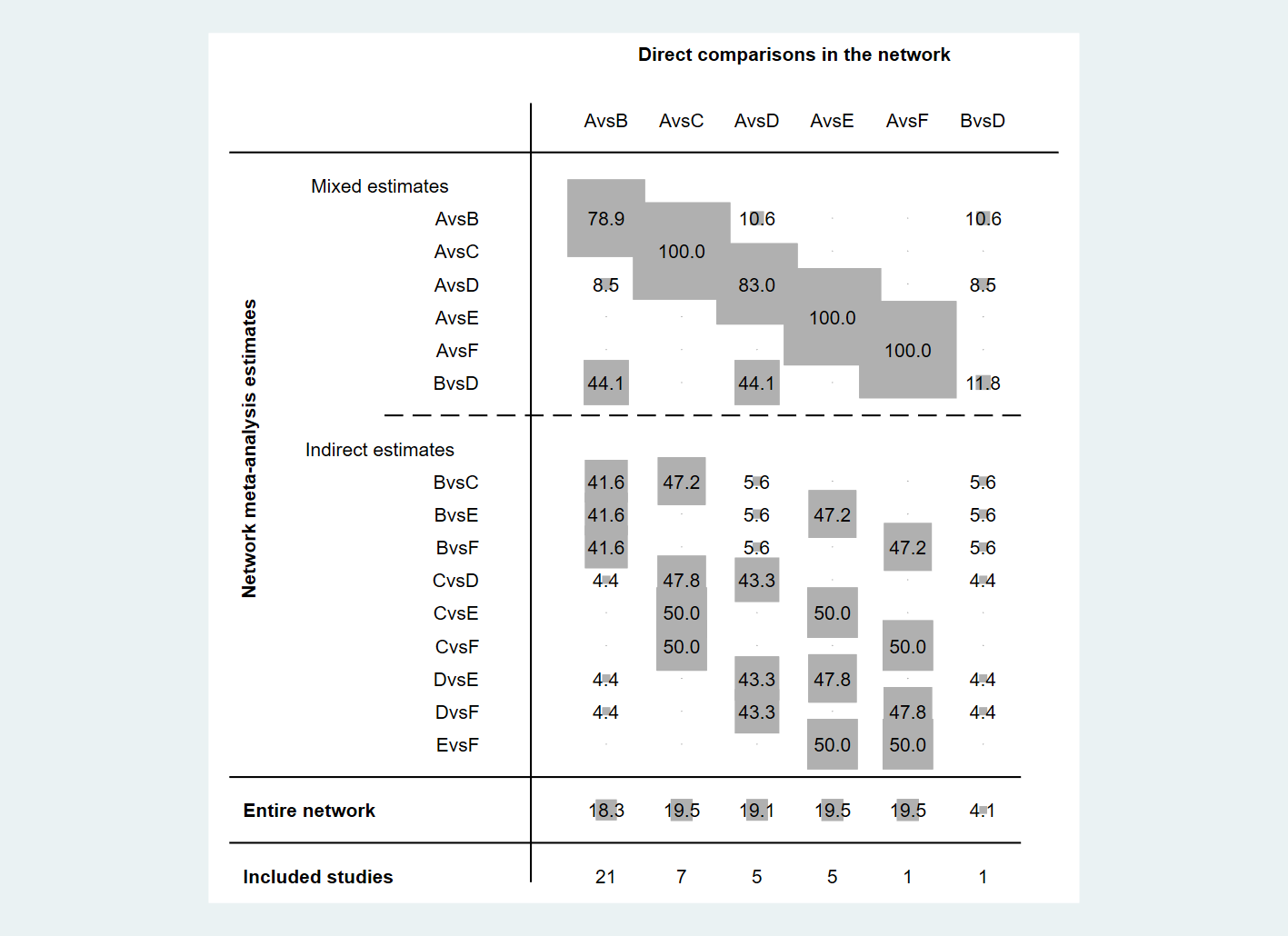


Figure S9 The Contribution of Depression Graph of the old adults

**Table S2 Subgroup analysis of mind-body exercise intervention for anxiety**

| Variable | Subgroup | Literature Count | SMD, CI | P-value | I^2^ |
| --- | --- | --- | --- | --- | --- |
| Health condition. | Health | 9 | -0.91 [-1.47, -0.35] | p$=0.001$ | 89% |
|  | anxiety | 4 | -0.78 [-2.05, 0.48] | p$=0.23$ | 98% |
| Age | ≤70 | 11 | -1.00[-1.63, - 0.37] | p$=0.002$ | 95% |
|  | >70 | 2 | -0.16 [-0.69, 0.37] | P=0.55 | 35% |
| Experimental group intervention measures | Tai Chi | 9 | -0.93 [-1.78, 0.08] | p$=0.03$ | 96% |
|  | Qi Gong | 2 | -0.85 [-1.79, 0.09] | P=0.08 | 84% |
|  | Yoga | 2 | -0.53 [-0.84, -0.21] | p$=0.001$ | 0% |
| Control group intervention measures | RDA | 2 | -1.96 [-5.03, 1.12] | p$=0.21$ | 98% |
|  | HE | 4 | -0.35 [-0.80, 0.10] | p$=0.13$ | 70% |
|  | UC | 5 | -0.54 [-0.97, -0.11] | p$=0.01$ | 64% |
|  | PE | 2 | -1.46 [-3.31, 0.40] | p$=0.12$ | 99% |
| Measures scale | SAS | 4 | -1.38 [-2.39, -0.37] | p$=0.007$ | 95% |
|  | HADS | 5 | -0.41 [-0.61, -0.21] | p$<$0.0001 | 0% |

Note: RDA= routine daily activities, HE= Health Education, UC= usual care, PE= physical exercise, SAS= Self-rating Anxiety Scale, HADS= Hospital Anxiety and Depression Scale

**Table S3 Subgroup analysis of mind-body exercise intervention for depression**

| Variable | Subgroup | Literature Count | SMD, CI | P-value | I^2^ |
| --- | --- | --- | --- | --- | --- |
| Health condition. | Health | 23 | -0.47 [-0.66, -0.29] | p$<$0.00001 | 65% |
|  | Depression | 15 | -0.67 [-1.04, -0.31] | p$=0.0003$ | 87% |
| Age | ≤70 | 23 | -0.56 [-082, -0.30] | p$<$0.0001 | 82% |
|  | >70 | 15 | -0.54[-0.80, -0.29] | p$<$0.0001 | 76% |
| Experimental group intervention measures | Tai Chi | 21 | -0.66 [-0.95, -0.36] | p$<$0.0001 | 87% |
|  | Qi Gong | 7 | -0.38 [-0.65, -0.11] | p$=0.005$ | 53% |
|  | Yoga | 5 | -0.52 [-0.78, -0.26] | p$<$0.0001 | 7% |
|  | Dance | 5 | -0.15 [-0.65, 0.36] | P$=0.57$ | 62% |
| Control group intervention measures | UC | 13 | -0.71 [-1.06, -0.36] | p$<$0.0001 | 77% |
|  | RDA | 4 | -0.65 [-1.07, -0.24] | p$=0.002$ | 73% |
|  | PE | 7 | -0.60 [-1.23, 0.03] | p$=0.06$ | 94% |
|  | HE | 4 | -0.61 [-0.85, -0.38] | P$<$0.00001 | 0 % |
|  | CT | 5 | -0.38 [[-0.68, -0.08] | P$=0.01$ | 16% |
|  | WL | 2 | 0.46 [-0.06, 0.99] | P$=0.08$ | 0% |
| Measures scale | GDS | 12 | -0.62 [-0.93, -0.31] | p$<$0.0001 | 78% |
|  | SDS | 3 | -1.04 [-1.86, -0.21] | p$=0.01$ | 86% |
|  | CES-D | 5 | -0.40 [-0.78, -0.03] | p$=0.04$ | 58.5% |
|  | HADS | 3 | -0.46 [-0.75, -0.16] | P$=0.002$ | 0.0% |
|  | HAMD | 4 | -0.50[-0.83, -0.16] | P$=0.004$ | 51% |
|  | BDI | 4 | -0.18 [-0.50, 0.15] | P$=0.30$ | 25% |

Note: UC= usual care, RDA= routine daily activities, HE= Health Education, PE= physical exercise, CT = cognitive training, WL= waiting list, GDS= Geriatric Depression Scale, SDS= Self-rating depression Scale, CES-D= Center for epidemiologic studies depression scale., HADS= Hospital Anxiety and Depression Scale HAMD= Hamilton Rating Scale of Depression, BDI= Beck Depression Inventory.

**Table S4 Meta-regression analysis of the impact of different moderator variables on anxiety**

| **Adjustable variable** | **Coef.** | **Std. Err** | **t** | **p>t** | **95%CI** |
| --- | --- | --- | --- | --- | --- |
| Age | 0.8284 | 0.7971 | 1.04 | 0.312 | -0.9260, 2.5829 |
| Health status | 0.1411 | 0.2831 | 0.50 | 0.627 | -2.2662, 0.7578 |
| Experimental group intervention measures | 0.1270 | 0.7869 | 1.61 | 0.115 | -0.0324, 0.2865 |
| Control group intervention | 0.1164 | 0.6158 | 1.89 | 0.067 | -0.0087, 0.2419 |
| Measures scale | 1.0120 | 0.3869 | 2.62 | 0.035* | 0.0973, 1.9268 |

**Table S5 Meta-regression analysis of the impact of different moderator variables on depression**

| **Adjustable variable** | **Coef.** | **Std. Err** | **t** | **p>t** | **95%CI** |
| --- | --- | --- | --- | --- | --- |
| Age | 0.0054 | 0.1947 | 0.03 | 0.978 | -0.3894, 0.4002 |
| Health status | 0.1928 | 0.1928 | 1.00 | 0.324 | -0.1982, 0.5839 |
| Experimental group intervention measures | 0.1270 | 0.0787 | 1.61 | 0.115 | -0.0324, 0.2865 |
| Control group intervention | 0.1166 | 0.0616 | 1.89 | 0.067 | -0.0087, 0.2419 |
| Measures scale | 0.0901 | 0.0645 | 1.49 | 0.147 | -0.3375, 0.2139 |

**Table S6 Risk of bias assessment**

| Study | Random sequence generation | Allocation concealment | Blinding of participants and personnel | Blinding of outcome assessors | Incomplete outcome data | Selective reporting | Other biases |
| --- | --- | --- | --- | --- | --- | --- | --- |
| Babaei Bonab S 2022 | low | unclear | unclear | unclear | low | unclear | low |
| Ge Y2022 | low | low | low | low | low | low | low |
| Song J 2022 | low | low | low | low | low | unclear | low |
| Redwine LS 2020 | low | unclear | unclear | unclear | low | low | low |
| Hsu CY2016 | low | unclear | unclear | unclear | low | low | low |
| Huang N2019 | low | low | unclear | unclear | low | unclear | low |
| Chou KL2004 | low | unclear | low | unclear | low | unclear | low |
| Ma C2018 | low | low | unclear | unclear | low | unclear | low |
| Leung RW 2012 | low | low | low | low | low | low | low |
| Yeh GY 2020 | low | unclear | unclear | unclear | low | unclear | low |
| Study | random sequence generation | allocation concealment | blinding of participants and personnel | blinding of outcome assessors | incomplete outcome data | selective reporting | other biases |
| Solianik R2021 | low | unclear | unclear | unclear | low | low | low |
| Lavretsky H2011 | low | low | low | unclear | low | unclear | low |
| Irwin MR 2014 | low | low | low | low | low | low | low |
| Li Y2019 | low | low | unclear | low | low | unclear | low |
| Lam L2014 | low | unclear | unclear | unclear | low | unclear | low |
| Yıldırım P 2015 | low | unclear | low | unclear | low | unclear | low |
| Noradechanunt C 2016 | low | low | low | unclear | low | unclear | low |
| Lavretsky H2021 | low | unclear | low | low | low | unclear | low |
| Chan AW2017 | low | low | unclear | low | low | unclear | low |
| Yeh GY2012 | low | unclear | unclear | unclear | high | unclear | low |
| Kilpatrick LA2022 | low | unclear | unclear | unclear | high | low | low |
| Study | random sequence generation | allocation concealment | blinding of participants and personnel | blinding of outcome assessors | incomplete outcome data | selective reporting | other biases |
| Liu J2018 | low | unclear | unclear | unclear | low | unclear | Low |
| Tsang HW2013 | low | low | unclear | low | low | unclear | low |
| Lee P2019 | low | low | unclear | unclear | low | unclear | low |
| Sun P2022 | low | unclear | unclear | unclear | unclear | unclear | low |
| Carcelén-Fraile MDC2022 | low | low | unclear | unclear | low | low | low |
| Jing L2018 | low | unclear | unclear | unclear | low | unclear | low |
| Martínez N2014 | low | unclear | low | low | low | unclear | low |
| Zhang YX2022 | low | unclear | unclear | unclear | low | unclear | low |
| Roswiyani R2022 | low | unclear | unclear | unclear | low | unclear | low |
| Haboush A2006 | low | unclear | unclear | unclear | low | unclear | low |
| Study | random sequence generation | allocation concealment | blinding of participants and personnel | blinding of outcome assessors | incomplete outcome data | selective reporting | other biases |
| Hashimoto H 2015 | low | unclear | low | unclear | low | unclear | low |
| Lee HJ2017 | low | unclear | low | low | low | unclear | low |
| Mavrovouniotis FH2008 | low | unclear | unclear | unclear | high | unclear | low |
| Ventura MI2016 | low | unclear | low | unclear | low | unclear | low |
| Choi MJ | low | unclear | unclear | low | low | unclear | low |
| Chang J 2021 | low | unclear | unclear | low | low | unclear | low |
| Eyre HA 2018 | low | low | low | high | low | low | low |
| Brian Frye 2007 | low | unclear | unclear | unclear | low | unclear | low |
| Krause-Sorio B2020 | low | unclear | unclear | unclear | low | unclear | low |
| Kwok JYY2019 | low | low | unclear | unclear | low | unclear | low |
| Curi VS 2018 | low | unclear | unclear | low | low | unclear | low |
